# An assessment of synthetic data generation, use and disclosure under Canadian privacy regulations

**DOI:** 10.1007/s43681-025-00819-0

**Published:** 2025-08-28

**Authors:** Lisa Pilgram, Anita Fineberg, Elizabeth Jonker, Khaled El Emam

**Affiliations:** 1 Children’s Hospital of Eastern Ontario Research Institute, Ottawa, Canada; 2 Department of Nephrology and Medical Intensive Care, Charite - Universitaetsmedizin Berlin, Berlin, Germany; 3 School of Epidemiology and Public Health, University of Ottawa, Ottawa, Canada; 4 Chang School of Continuing Education, Toronto Metropolitan University, Toronto, Canada; 5 Anita Fineberg, LL.B., CIPP/C, Toronto, Canada

**Keywords:** Synthetic data, Privacy, Disclosure risk, Generative artificial intelligence

## Abstract

Synthetic data generation (SDG) plays an increasingly important role as a research and innovation accelerator. While SDG can enable privacy-preserving data sharing, it also raises privacy concerns compounded by uncertainty how privacy law applies to SDG and the generated data itself. Such uncertainty can hinder positive applications of SDG and put individual privacy rights at risk. This study aims to understand how SDG and synthetic data are treated under Canadian federal privacy law, identifying regulatory gaps that extend beyond the Canadian context and proposing recommendations to address them. Our analysis shows that SDG is not explicitly addressed by the statute. While SDG arguably qualifies as a use of personal information, it is unclear whether consent is required for SDG. Further Fair Information Practices with respective obligations apply to SDG just as they do to any use of personal information. The generated data itself could fall outside the law’s scope since it is more likely to qualify as non-personal than traditionally de-identified data but the concept of identifiability under the statute remains ambiguous, particularly regarding inferences. An unclear definition of identifiability represents a relevant gap in privacy law that can harm the individual directly, through the exposure of personal information, or indirectly, by hindering the adoption of SDG and other beneficial privacy-enhancing technologies. A Code of Practice, anchored in legislation, could address such privacy concerns and ensure the proper application of SDG.

## Introduction

Interest in and adoption of synthetic data generation (SDG) has been growing quite rapidly over the last few years [1–3]. Synthetic data can serve multiple purposes across sectors: it can serve privacy use cases by enabling data sharing, it can serve de-biasing use cases by compensating for representation bias that is present in data, and can serve augmentation use cases by building reliable models from small sample sizes [2, 4]. In a health data context, for example, SDG as enabler of privacy-preserving data sharing could be useful for secondary research, algorithm testing, health IT development and education [5, 6]. This includes data exploration and understanding in the research phase, software testing in the development phase, and demonstrations or educational training in the integration phase [7]. Another example is fraud detection in the financial sector, whereby SDG creates realistic transaction data that can be used to develop relevant software without exposing personal customer information [8], or synthetic urban mobility data that can be leveraged to support traffic management and smart city development while preserving privacy [9].

At the same time, there are two major concerns around SDG that are also recurrent topics in the artificial intelligence (AI)/machine learning (ML) and big data literature: bias and privacy [10–13]. The privacy concerns are the ones of interest for this article, particularly as they relate to the question of regulatory oversight.

###  Synthetic data generation

There are multiple ways that synthetic data can be generated. One approach is using distributions known a-priori and informed by background knowledge, published summary statistics, and published risk calculators [14–18]. However, SDG is mostly achieved by training a generative AI/ML model on real data and then sampling the synthetic data from that trained model [19]. The resulting synthetic dataset should mimic the characteristics of the real data but does not contain records that pertain to actual individuals.

The term AI/ML refers to computational models that learn from data rather than relying on fixed, rule-based formulas. There is a large variety of models that can be leveraged for SDG such as sequential trees or generative adversarial networks [2]. The common concept is that the model learns the underlying distribution (or patterns) of real data during training, essentially capturing a high-level representation of that data. Once trained, the model can generate synthetic data by sampling from that distribution. This synthetic data mimics the statistical properties of the real data. Similar to prediction models, the goal during training is to develop a model that generalizes well rather than overfitting to the real data. This means that it should not be too tailored to the real data (i.e., overfitting) but also perform well on new, unseen data. Most models have therefore some integrated regularization mechanisms to prevent overfitting and ensure better generalization.

The resulting synthetic dataset is typically evaluated in terms of fidelity, utility and privacy [20]. Fidelity measures the resemblance or similarity between the synthetic data and the original (real) data [20–23]. Downstream utility assesses how well synthetic data performs in a specific downstream task. This gives valuable insights into the functionality of synthetic data in practice and can, for example, be an analytical workload, such as statistical inference [24] or prediction [25]. Privacy evaluation aims to assess whether the synthetic data may still expose personal information [20, 23, 26–28]. Importantly, all three dimensions typically require access to the original (real) data for meaningful evaluation.

Among the various contemporary privacy enhancing technologies (PETs), SDG has the unique feature that, in principle, records in the resulting dataset have no one-to-one mapping to records in the training dataset. This protects individuals from identity disclosure by design. Accordingly, one might be tempted to say that privacy regulations do not apply to synthetic data. But practical experiences suggest that there can be residual vulnerabilities in synthetic data. For example, reproduction of records can still happen by chance or due to overfitting of the generative models, and it has been shown that synthetic data is also vulnerable to inference attacks, such as membership and attribute disclosure [29–32].

###  Regulatory aspects of synthetic data generation

While the potential privacy benefit of synthetic data is widely recognized, it remains a relatively new and evolving technology with regulatory uncertainties: very few data protection authorities have published SDG guidelines [33–35], synthetic data is not explicitly mentioned in most existing privacy laws, and actual court cases providing precedents are lacking. However, more recent laws outside the privacy domain such as the European AI Act [36] and the Utah AI Policy Act [37] have started to explicitly acknowledge synthetic data as PET.

Opinions on SDG under the European General Data Protection Regulation (GDPR) typically treat SDG as another (novel) technique of de-identification [38–43]. The European Data Protection Supervisor (EDPS) is in line with such an interpretation [44] and further expressed optimism in using SDG as a privacy- and utility-preserving technology [45].

The process of generating synthetic data is generally seen as processing personal information, requiring a lawful basis under the GDPR, while resulting synthetic data may qualify as non-personal information. A lawful basis may be consent, but legitimate interest can also be claimed whereby SDG can be a relevant argument in the balancing test [19]. Legitimate interest arguments always require the data controller to balance their legitimate interest against the risk to the data subjects. For example, scientific purposes or marketing and advertising are listed in the Working Party 29 opinion as legitimate interests [46]. If an organization wants to use synthetic data for such purposes and SDG occurs within a trusted execution environment, then the risk to data subjects is arguably small and does not appear to override the legitimate interests of the organization. A lawful basis does not, however, free the organization from other legal obligations such as fairness, proportionality and transparency.

In the United States (US), the situation is more complex. Synthetic data may, for example, fall outside the scope of the Children’s Online Privacy Protection Act (COPPA) as the law only applies to personal information being collected online, while synthetic data is generated [47]. Under the California Consumer Privacy Act (CCPA), synthetic data is also not a term used in the statute but can either be seen as de-identified data that is defined as information with no reasonable possibility to be linked to an individual, or similar to aggregate information. Both are explicitly outside the scope of CCPA [19, 47, 48]. And under the Health Insurance Portability and Accountability Act (HIPAA), de-identified information that meets specified criteria is not considered individually identifiable health information (i.e., protected health information). SDG can result in such data that would then fall outside the statute’s scope [19, 47].

Despite the uncertainty around the regulatory environment, synthetic data is being made publicly available, such as cancer data from Public Health England [49], synthetic variants of the French public health system claims and hospital dataset [50], and synthetic microdata from Israel’s National Registry of Live Births [51]. Also, authors have started to share their synthetic data alongside their research papers in multiple jurisdictions including Europe and Canada [52–55].

As synthetic data becomes more publicly available, clear regulatory frameworks are needed. Gaps in privacy regulation can result in privacy risks, which means that they can impact individuals’ privacy rights, or that they constitute barriers to the adoption of SDG [56] which, in turn, can result in the use of personal information instead.

###  The Canadian regulatory context

There have not been analyses on how synthetic data is regulated in a Canadian context. In this paper, we analyze how the current federal private sector privacy law in Canada (i.e., Part I of the Personal Information Protection and Electronic Documents Act (PIPEDA) [57]) regulates generating synthetic data, as well as how it regulates the use and disclosure of the generated synthetic data itself.

The regulatory landscape in Canada is divided between federal and provincial governments. Federal legislation such as PIPEDA applies to federally regulated organizations and to provincially regulated organizations if the organization is engaged in commercial activities and the provincial private sector legislation has not been deemed to be substantially similar to PIPEDA or no provincial private sector legislation has been enacted.

PIPEDA anchors its statutory purpose in establishing “rules to govern the collection, use and disclosure of personal information” [57]. These three processes are important components of the broader data lifecycle [58]. However, PIPEDA is largely technology-agnostic and provides limited definitions on relevant terms, including the very lifecycle processes it aims to regulate. Consequently, uncertainty remains about how the law applies to evolving data practices such as the generation, use and disclosure of synthetic data.

This legal ambiguity underscores the need for a comprehensive analysis of SDG, synthetic data use and disclosure under PIPEDA. In this paper, we highlight areas where key points are open for interpretation, offer concrete recommendations to close these gaps, and discuss common gaps when interpreting and applying Canadian, but also international, privacy law. This is particularly timely given that there have been ongoing efforts to update the current Canadian federal privacy law (e.g. Bill C-27) [59] and that the Canadian province of Quebec has just recently enacted a new privacy law [60] with the possibility of other provinces or territories following. Learnings from this analysis can guide regulators worldwide in considering relevant factors when regulating AI-driven privacy-enhancing technologies.

### Questions on regulating synthetic data generation, use and disclosure

**Fig. 1:**
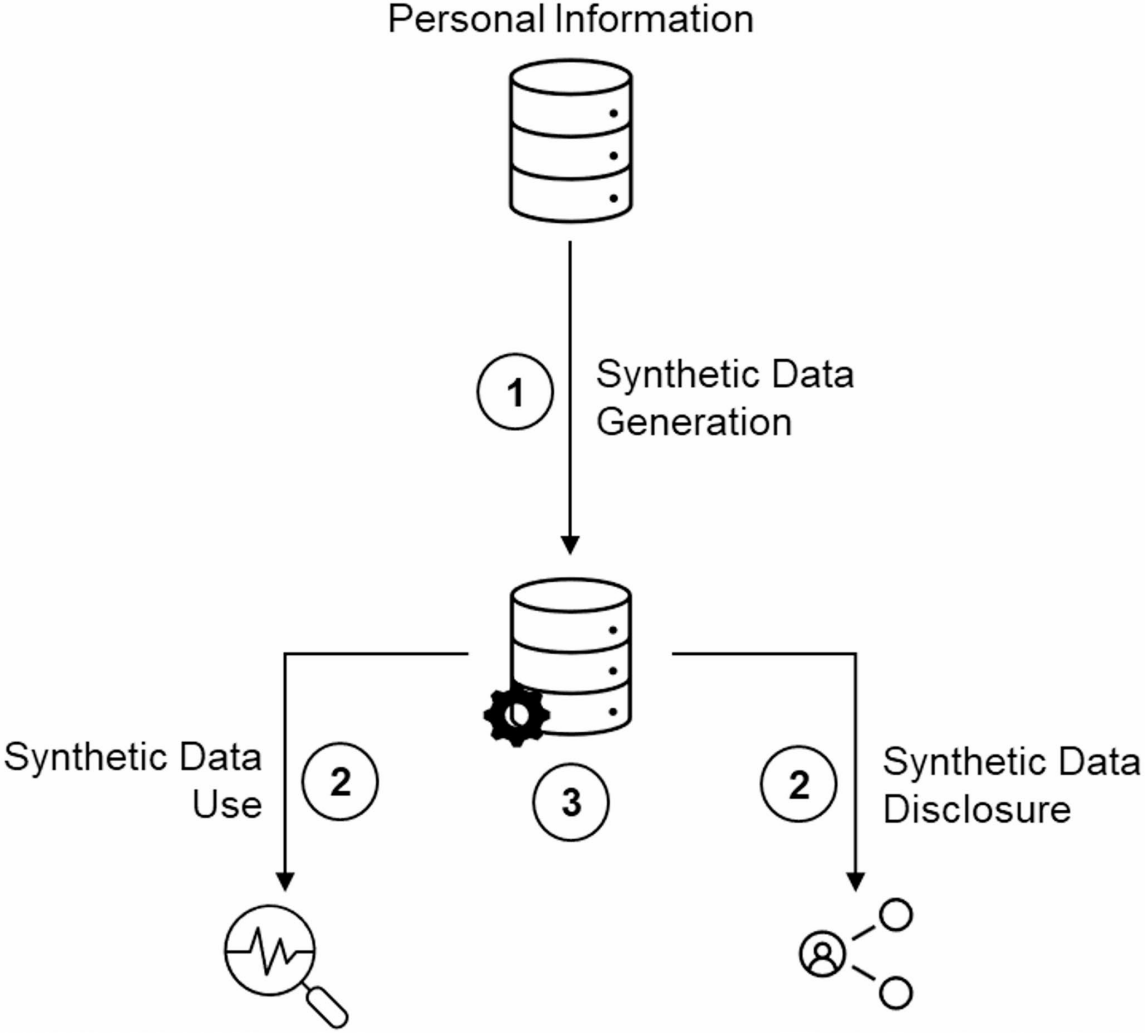
Synthetic Data Generation, Use and Disclosure. The indicated numbers correspond to the questions asked in this study

To assess the treatment of synthetic data under PIPEDA, we adopt a question-based approach that reflect three relevant processes in the synthetic data’s entire lifecycle, namely its generation, use and disclosure (see Fig. 1).

These processes have been relevant in evaluations of other legal frameworks as well [19, 38–41] reflecting core challenges that have emerged globally in the legal discourse around SDG as a PET. They do, however, not precisely map to the statutory language of PIPEDA. PIPEDA, as previously mentioned, approaches the regulation of personal information primarily through the lens of collection, use and disclosure. The ten Fair Information Principles (FIPs), incorporated into PIPEDA through Schedule 1, extend this scope to include additional lifecycle processes such as access and retention.

To assess both the relevance of SDG, synthetic data use and disclosure as well as the purpose of PIPEDA, we structure our analysis around three main questions. These are further broken down into six specific questions, selected to reflect common considerations under other legal frameworks [19, 38–41] and to address practical considerations that frequently arise in real-world SDG implementations. For example, Question 1c reflects the practical observation that SDG is increasingly offered by specialized providers (e.g. MDClone [61] or MOSTLY AI [62]) which creates scenarios where organizations may outsource the process to third-party service providers. In such cases, SDG is often performed in the provider’s cloud environment, requiring a transfer of personal information so that the provider can conduct SDG on the organization’s behalf.

The six questions are as follows:

1. Is the use of the original (real) dataset to generate or evaluate a synthetic dataset regulated?
  - Is consent required, or is there another valid legal basis for processing personal information for SDG?
  - What are the obligations of organizations to be transparent with respect to their processing of personal information for SDG?
  - Is sharing of the original data set by an organization with a third-party service provider to generate the synthetic data set regulated?
2. Does the statute regulate the resulting use and disclosure of the synthetic data set?
3. What is the impact of the creation, use and disclosure of synthetic data on specific individual privacy rights, such as the right to the withdrawal of consent, access, correction and deletion?

An important assumption in our analysis is that the original (real) dataset used for SDG qualifies as personal information within the meaning of the statute. This is a necessary starting point for analyzing these questions under PIPEDA, as the statute applies only to personal information. If the original (real) dataset were not considered personal information, the questions above would not be triggered. We further assume that the organization lawfully holds the original (real) dataset, for example, through data collection or through a contractual arrangement. While the collection of personal information is a process regulated under PIPEDA, it is relatively well-established in practice and not directly relevant to SDG, its use and disclosure.

Each of the six questions is addressed through an interpretation of PIPEDA and supported where appropriate by emerging opinions from other jurisdictions. This allows for broader legal reasoning where interpretation of PIPEDA remains limited. Actual court cases with precedents with respect to synthetic data are lacking so that we could not leverage case law for our analysis. We provide responses to these questions and describe identified gaps and resulting risks. Recommendations are proposed on how these gaps may be addressed to encourage positive applications of synthetic data while at the same time protecting individual privacy rights.

##  Analysis of synthetic data under PIPEDA

###  Is the use of the original (real) dataset to generate and/or evaluate a synthetic dataset regulated?

This main question starts by first determining whether the use of personal information for SDG and synthetic data evaluation falls within the scope of PIPEDA. If so, three sub-questions then examine relevant legal implications, namely the requirements for consent, transparency, and whether such processing activities can be outsourced to third-party service providers.

It is not explicitly clear whether the use of the original data (real personal information) to generate and/or evaluate a synthetic data set is regulated under PIPEDA. This stems from the absence of SDG as a term that is directly used in PIPEDA. SDG can be described as a technology to reduce the identifiability of data [38]. Other processes or technologies that aim at reducing the identifiability of the data are pseudonymization, de-identification, or anonymization. These technologies typically start with personal information as input and output data of reduced identifiability. Some jurisdictions explicitly define such technologies as a form of “use” or “processing” personal information [63]. Defining specific technologies in PIPEDA would not be in line with its technology-neutral approach. However, it also does not refer to the process of reducing the identifiability of data as a regulated activity through which an organization can “interact” with personal information which is, for example, the case in the Ontario *Personal Health Information Protection Act, 2004* (PHIPA) [64]. Instead, PIPEDA refers more broadly to any such interactions under the general category of “use” of personal information.

While the absence of a definition of the term “use” in PIPED leaves it open to interpretation whether or not SDG or other related technologies such as pseudonymization, de-identification, or anonymization are formally included under the term, there is likely little dispute that personal information is in fact “used” in these processes, particularly when considering how “use” is defined in other Canadian privacy legislation. For example, Ontario’s PHIPA defines “use” as “view, handle or otherwise deal with the information” [64]. The same applies to the evaluation of synthetic data that typically involves the (real) training data (i.e., personal information).

### Is consent required, or is there another valid legal basis for processing personal information for SDG?

Assuming that SDG and evaluation fall within the scope of “use” under PIPEDA, the next question is how is it regulated? One important consideration is the legal basis for processing personal information for SDG. Schedule 1 of PIPEDA contains 10 principles (i.e., FIPs) as part of the regulatory framework for how organization must handle personal information. As personal information is arguably “used” in SDG, the Schedule 1 would then be as a whole applicable to SDG. Its Principle 3—Consent (Section 4.3  of Schedule 1) makes clear that PIPEDA treats consent as the default legal basis, more precisely, it states that “The knowledge and consent of the individual are required for the collection, use, or disclosure of personal information, except where inappropriate.”.

Subsection 7 (2) sets out explicit instances in which an organization may use personal information without the knowledge or consent of the individual to whom the information relates (see sidebar). None of the listed instances may be interpreted so as to include the use of personal information to generate synthetic data or, more broadly, for the purpose of reducing the identifiability of the data, and to evaluate this process.

**Table:** 

Sidebar: Use of personal information without the knowledge or consent under PIPEDA			
(7)(2) For the purpose of clause 4.3 of Schedule 1, and despite the note that accompanies that clause, an organization may, without the knowledge or consent of the individual, use personal information only if			
(a)		in the course of its activities, the organization becomes aware of information that it has reasonable grounds to believe could be useful in the investigation of a contravention of the laws of Canada, a province or a foreign jurisdiction that has been, is being or is about to be committed, and the information is used for the purpose of investigating that contravention;	
(b)		it is used for the purpose of acting in respect of an emergency that threatens the life, health or security of an individual;	
		(b.1) the information is contained in a witness statement and the use is necessary to assess, process or settle an insurance claim;	
		(b.2) the information was produced by the individual in the course of their employment, business or profession and the use is consistent with the purposes for which the information was produced	
(c)		it is used for statistical, or scholarly study or research, purposes that cannot be achieved without using the information, the information is used in a manner that will ensure its confidentiality, it is impracticable to obtain consent and the organization informs the Commissioner of the use before the information is used;	
		(c.1) it is publicly available and is specified by the regulations; or	
(d)		it was collected under paragraph	
		(1)(a) the collection is clearly in the interests of the individual and consent cannot be obtained in a timely way;	
		(1)(b) it is reasonable to expect that the collection with the knowledge or consent of the individual would compromise the availability or the accuracy of the information and the collection is reasonable for purposes related to investigating a breach of an agreement or a contravention of the laws of Canada or a province	
		(b.01) it was disclosed under paragraph (3)(d.21)	
		(1)(e) the collection is made for the purpose of making a disclosure (i) under subparagraph (3)(c.1)(i) or (d)(ii) or (ii) that is required by law	
The subparagraphs (3)(c.1)(i) and (d)(ii) allows disclosure of personal information without consent of the individual if the disclosure is made to a government institution or part of a government institution when suspecting that the information relates to national security, the defence of Canada or the conduct of international affairs			

Thus, it is not clear as to whether an organization may use personal information for the purposes of creating and evaluating a synthetic data set in the absence of an individual’s knowledge or consent. From a strict legal interpretation perspective, the case can be made that because subsection 7 (2) of PIPEDA already sets out the circumstances in which knowledge and consent are not required, one cannot “read in” any additional uses for which individual consent is not required. The list of exceptions is finite, and the opening language provides that “only if” the listed circumstances apply, knowledge and consent are not required.

Among these limited exemptions, however, paragraph 7(2)(c) permits the use of personal information without consent for statistical or scholarly study or research purposes, if certain conditions are met (see sidebar). One of these conditions is that “the information is used in a manner that will ensure its confidentiality”. SDG is arguably a technology that serves as a mechanism for ensuring confidentiality, so that SDG might not only be permissible under paragraph 7(2)(c) but could also help to satisfy the confidentiality requirement.

More broadly, it can also be argued that when the use of the personal information is for privacy-protective purposes that may benefit an individual, their knowledge and consent should not be required. There are anecdotal reports that lawyers have interpreted PIPEDA from a “purpose-based” vs. “strict legal interpretation” perspective. They conclude that because the objective of using and evaluating a PET is to protect individual privacy while maintaining the utility of the data for secondary purposes, consent need not be required for two reasons. The first argument is about the individual’s privacy: if an organization needs to obtain consent for applying a PET to personal information, the organization may as well obtain consent to use the individual’s personal information directly for the secondary purpose instead. While organizations may have other motivations for implementing PETs, for example as a trust-building exercise or to align with corporate social responsibility goals, such an approach can reduce the incentive for organizations to adopt PETs [65]. Also, Principle 1—Accountability (Section 4.1 of Schedule 1) reinforces the implementation of procedures to protect personal information. SDG is arguably one of such procedures, contributing to an organization's broader accountability obligations. This aligns with the Office of the Privacy Commissioner of Canada (OPC) 2020 recommendation on the regulation of AI that organizations implement “appropriate technical and organizational measures” to meet PIPEDA requirements   [66].

The second argument is more fundamentally about the challenges of consent in practice. Critics have questioned whether current consent practices including the increasing adoption of broad consent models [67, 68] truly reflect an individual’s informed understanding of how their data will be used [69–71] which is a requirement under the Principle 3—Consent in Schedule 1 of PIPEDA. Even if consent practice was perfectly aligned with this Principle, there have been concerns about the negative impact of consent requirements on, for example, the validity of health research. A primary reason is that individuals who consent tend to be different to those who do not consent [68, 69, 72–76]. This is referred to as consent bias and can limit the generalizability of findings to the broader population. One example of consent bias is the phenomenon of “worried well” whereby individuals who are generally healthy, well-educated and from a higher socioeconomic background tend to have a greater willingness to consent [75, 76]. This can ultimately contribute to health inequalities.

### What are the obligations of organizations to be transparent with respect to their processing of personal information for SDG?

The other FIPs in Schedule 1 are framed as organizational obligations and one important question is whether an organization has an obligation to be transparent about their use of personal information for the purposes of creating a synthetic dataset according to the Schedule.

“Transparency” from a practical perspective is to provide individuals with information about how the organization processes personal information.

In this sense, the Principle 3—Consent is arguably a very straightforward form of transparency under PIPEDA as it is inherently linked to the individual’s knowledge of the purposes for which their personal information is being used or disclosed. In practice, as discussed in the previous question, there are serious concerns whether current consent practices truly reflect an individual’s informed understanding of the purposes [69–71]. If this is not the case, consent can in fact decrease transparency by obscuring rather than clarifying how personal information is handled. Beyond consent, transparency is typically meant more as a notice function: the obligation to proactively inform individuals about privacy practices as anchored in various FIPs.

These include Principle 1—Accountability, Principle 2—Identifying Purposes, Principle 4—Limiting Collection, Principle 5—Limiting Use, Disclosure, and Retention and Principle 8—Openness. Under Principle 1, organizations are required to communicate “The identity of the individual(s) designated by the organization to oversee the organization’s compliance with the principles shall be made known upon request” (Section 4.1.4(d) of Schedule 1). Principle 2 and Principle 4 are primarily related to the collection of personal information; they extend, however, beyond the collection and remain applicable when using the collected personal information. More precisely, Principle 2 requires that organizations “shall document the purposes for which personal information is collected in order to comply with the Openness principle (Clause 4.8) and the Individual Access principle (Clause 4.9)” (Section 4.2.1 of Schedule 1) and “be able to explain to individuals the purposes for which the information is being collected” (Section 4.2.5 of Schedule 1). Under Principle 4, organizations “shall specify the type of information collected as part of their information-handling policies and practices, in accordance with the Openness principle (Clause 4.8)” (Section 4.4.1 of Schedule 1). Principle 5 reinforces that these requirements carry over to the use and disclosure of personal information as it “shall not be used or disclosed for purposes other than those for which it was collected, except with the consent of the individual or as required by law” (Section 4.5 of Schedule 1). Ultimately, the Principle 8—Openness requires organizations to make “specific information about its policies and practices relating to the management of personal information” available to individuals (Section 4.8 of Schedule 1). This includes details such as the type of personal information held, the means of access, and whether it is shared with related organizations (Section 4.8.2 of Schedule 1).

These provisions are relevant to transparency and would apply to SDG as to any other use of personal information. Also, as mentioned previously, Principle 3—Consent is foundational to transparency. But even where consent is not legally required, the above referenced principles would still apply.

### Is sharing of the original data by an organization with a third-party service provider to generate the synthetic dataset regulated?

To carry out the process of SDG, organizations may rely on third-party service providers. Such providers offer technical expertise, resources and/or proprietary tools for SDG.

PIPEDA provides information about the sharing of personal information with a third-party service provider. The legal framework for the sharing of personal information by an organization with a third-party service provider is set out as follows in Schedule 1 of PIPEDA under Principle 1—Accountability (see Table 1).

**Table 1:** Accountability principle and provisions according to PIPEDA Schedule 1

Principle	Provisions in Schedule 1 to PIPEDA	
	Section #	Obligation
Accountability	4.1	An organization is responsible for personal information under its control and shall designate an individual or individuals who are accountable for the organization’s compliance with the following principles
	4.1.1	Accountability for the organization’s compliance with the principles rests with the designated individual(s), even though other individuals within the organization may be responsible for the day-to-day collection and processing of personal information. In addition, other individuals within the organization may be delegated to act on behalf of the designated individual(s)
	4.1.2	The identity of the individual(s) designated by the organization to oversee the organization’s compliance with the principles shall be made known upon request
	4.2.3	An organization is responsible for personal information in its possession or custody, including information that has been transferred to a third party for processing. The organization shall use contractual or other means to provide a comparable level of protection while the information is being processed by a third party
	4.1.4	Organizations shall implement policies and practices to give effect to the principles, including (a) implementing procedures to protect personal information; (b) establishing procedures to receive and respond to complaints and inquiries; (c) training staff and communicating to staff information about the organization’s policies and practices; and (d) developing information to explain the organization’s policies and procedures

This principle introduces new terminology not used or defined in the legislation itself. This includes the term “third party” which is not further defined to encompass, for example, service providers, but also terms to define the activities involved in the data lifecycle. The key gaps in the terminology used in this Principle are those related to the meaning of “transferred” and “processing”. This is not only with respect to their meaning, but perhaps more importantly, the relationship between these new terms and those that are used in the Act itself, namely “collection”, “use” and “disclosure”.

Applying the terminology of Schedule 1 to the question posed: Is transferring of personal information by an organization to generate and evaluate a synthetic dataset (“processing” the personal information) regulated under PIPEDA? The OPC has confirmed that the term “transfer” “is a use by the organization. It is not to be confused with a disclosure. When an organization transfers personal information for processing, it can only be used for the purposes for which the information was originally collected” and further that “processing” “is interpreted to include any use of the information by the third-party processor for a purpose for which the transferring organization can use it.” [77].

Then, with SDG being a “use” and thereby a “processing” of personal information, the same considerations as discussed in the first questions apply. If it is a permitted use of personal information without consent by the data custodian, then so too would be the transfer of the original data set to a third-party service provider to use the personal information for SDG. However, in this scenario additional obligations would be imposed on the organization to “use contractual or other means to provide a comparable level of protection while the information is being processed by a third party”.

### Does the statute regulate the resulting use and disclosure of the synthetic dataset?

Synthetic data is the result of SDG. Other data categories that result from similar technologies are pseudonymized, de-identified, or anonymized data. Some jurisdictions explicitly define criteria for these data categories and corresponding obligations [63]. PIPEDA, however, does not define different data categories, so that this question can be put another way relating to the identifiability of the synthetic data—whether it may be characterized as personal information so that it falls within the scope of the statute. Therefore, the answer to the question depends on the interpretation of identifiability or personal information since identifiability leads to a consideration of whether legislation applies at all.

Personal information under PIPEDA is defined as “information about an identifiable individual” [57]. There are, however, no further details on what constitutes identifiability. This encompasses two important aspects of identifiability. First, there is the question of the nature of identifiability. While identity disclosure has traditionally been the focus of privacy regulation, recent literature and regulatory developments increasingly acknowledge that inferential disclosure may also render information personal [23, 28, 78, 79]. And second, since identifiability can be seen as a spectrum in practice [80, 81], there is the question of the degree or level of identifiability. That is, what level of identifiability is sufficiently low for data to fall outside the scope of the statute.

We discuss both aspects and examine how identifiability and data of different identifiability have been regulated in other Canadian and non-Canadian contexts before coming back to the question posed.

As mentioned above, privacy laws have defined terms for data of reduced identifiability such as pseudonymized, de-identified or anonymized data which are, however, inconsistent across jurisdictions [63]. According to the Government of Canada’s Personal Information and Privacy Glossary pseudonymized data is data where “the attributes that allow the direct identification of a person are replaced by pseudonyms” [82] and de-identification is defined by the Canadian Anonymization Network as a “process that transforms personal information into data for which there is no serious risk of re-identifying an individual given the context in which it will be processed” resulting in de-identified data [83]. The term anonymized data is not frequently used in the Canadian federal regulatory context. Sub-nationally, however, recent Canadian provincial privacy regulation in Quebec [60] defines anonymized data as being subject to three criteria (i.e., correlation, individualization and inference criterion—see Table 3) and requires that “it is at all times, reasonably foreseeable in the circumstances that the information produced further to a process of anonymization irreversibly no longer allows the person to be identified directly or indirectly”. They further clarify that this does not imply zero risk but requires a very low residual risk.

These definitions capture different aspects of identifiability: while pseudonymized and de-identified information address the level of identifiability, the definition of anonymized data in Quebec (see Table 3) [60] and in the European context (see Table 2) [84] also gives details on the nature of identifiability by introducing three explicit criteria.

**Table 2:** Risks Essential to Anonymization According to the Opinion 05/2014 by the Article 29 Protection Working Party [84]

	Risk definition
Singling Out	Which corresponds to the possibility to isolate some or all records which identify an individual in the dataset;
Linkability	Which is the ability to link, at least, two records concerning the same data subject or a group of data subjects (either in the same database or in two different databases). If an attacker can establish (e.g. by means of correlation analysis) that two records are assigned to a same group of individuals but cannot single out individuals in this group, the technique provides resistance against “singling out” but not against linkability;
Inference	Which is the possibility to deduce, with significant probability, the value of an attribute from the values of a set of other attributes

But outside of Quebec, the Canadian context has historically related identifiability only to the concept of identity disclosure [80, 85] which is also an interpretation of the criterion “singling out” or “linkability” of anonymized data [85, 86]. Identity disclosure is the idea of assigning a record to the corresponding identity of an individual. Yet, there are individual examples where regulators have considered broader notions of identifiability. In one investigation under PIPEDA, the OPC investigated a data-matching process that combined publicly available telephone directory listings with geo-demographic statistics [87]. Inferential disclosures, more precisely attribute disclosure (see below for the definition), were considered in the analysis and, while the concerns were ultimately not found to be valid, it demonstrates that Canadian federal regulators have in the past looked beyond identity disclosure alone.

The focus of disclosure risks with synthetic data, however, is increasingly on inferences, namely attribute and membership inference [23, 28, 78, 79]. Membership disclosure is concerned about inferring that an individual was in the training dataset for the SDG model (i.e., a member of the training dataset). Attribute disclosure has been defined as when an adversary can infer sensitive information about a target individual from the dataset’s attributes [88–90]. As discussed in the more technical privacy literature, correct inferences can occur independently of any individual’s data being disclosed. Therefore, both disclosure types are about the incremental inference that can be attributed to the dataset [31, 78, 91, 92]. A key question is whether an adversary can infer personal information about an individual that they would not have been able to learn if the individual had not been included in the dataset. And while early privacy models like l-diversity [93] lacked practical applicability, more recent work has introduced definitions of such inferential disclosure risks that are operationally feasible and better aligned with real-world risks [28]. Against this background, it becomes clear that one question is whether the concept of identifiability as it is defined in PIPEDA can be extended to cover inferential disclosures, as more recent privacy regulations in Europe and Quebec suggest (see Tables 2 and 3).

**Table 3:** Criteria applicable to anonymization according to the regulation respecting the anonymization of personal information in Quebec [60]

	Criterion definition
Correlation criterion	Means the inability to connect datasets concerning the same person;
Individualization criterion	Means the inability to isolate or distinguish a person within a dataset;
Inference criterion	Means the inability to infer personal information from other available information

The second important consideration is the degree of identifiability. Pseudonymized information as defined above, for example, can still contain indirect identifiers that have a high risk of identifying a specific individual and is therefore typically treated as personal information. In contrast, anonymized and de-identified data can have very low residual risk and, if below a specified threshold [85, 86], can qualify as non-personal.

While most privacy laws treat the identifiability of information as a binary construct: information is either personal or not, the underlying assessment of identifiability is typically probabilistic and contextual, so that account should be taken of all means reasonably likely to be used to identify the individual (“reasonably foreseeable”, “serious possibility”, “reasonably likely to be used”, “reasonably be expected”) [63, 94]. This implies that identifiability exists along a spectrum rather than as an absolute state which aligns more closely with practical privacy considerations [80, 81]. In contrast, the WP29 Opinion 05/2014 [84] as early guidance under the GDPR has been interpreted as implying an absolute zero-risk definition. This position is generally regarded as unrealistic in the real-world [95] and it has been challenged by international technical standards (e.g. ISO/IEC 27559:2022 [86]) and more recent regulatory guidelines (e.g. [33, 85]) that recognize that some very low residual risk is inevitable. Regulatory practice over the past decades has established relevant precedents for such values. For example, Health Canada recommends a threshold of 0.09 for de-identified health datasets [96] and the de-identification guidelines by the Information and Privacy Commissioner of Ontario gives a range between 0.1 and 0.05 depending on the sensitivity of the data [85]. Similarly, the International Standard on Information security, cybersecurity and privacy protection—Privacy enhancing data de-identification framework (ISO/IEC 27559) provides guidance on acceptable thresholds [86]. Importantly, these precedents have been typically applied in the context of identity disclosure.

Coming back to the question regarding the applicability of PIPEDA to synthetic data, the answer depends on how broadly identifiability can be interpreted under PIPEDA. One could make the case that identifiability has historically been mainly interpreted as identity disclosure under PIPEDA, and that synthetic data does not retain a link to the real record and consequently to the real identity by design. As such, synthetic data may fall outside the common interpretation of “personal information” under PIPEDA.

However, the more recent privacy regulations in Europe and Quebec have expanded the concept of identifiability to cover more than just traditional identity disclosure. And because such inferential disclosures may still be possible from synthetic data, it would be reasonable to assume that they must be considered in its identifiability assessment. This means that a synthetic dataset can reveal personal information without necessarily exposing an identity (i.e., identity disclosure). If it is demonstrated that the inferential disclosures from synthetic data are very small, then the synthetic data would be non-personal. The mechanics of how this can be verified is beyond the scope of this study but, nonetheless, a recurrent topic when discussing the legal status of synthetic data [38–41].

Yet, even if inferential disclosures were clearly accepted as relevant under PIPEDA, the statute offers no guidance on what level of residual risk is permissible. And while precedents exist for identity disclosure, similar thresholds for inferential disclosures are lacking. This absence of clear guidance adds to the uncertainty in answering the question of whether synthetic data can be considered non-personal and thereby fall outside the scope of the statute.

### What is the impact of the creation, use and disclosure of synthetic data on specific individual privacy rights, such as the right to the withdrawal of consent, access, correction and deletion?

Under PIPEDA, individual rights are grounded in Schedule 1 which provides a framework under which organizations must operate when handling personal information. While the principles in Schedule 1 are framed as organizational obligations, several imply corresponding individual privacy rights. More precisely, the Principle 3—Consent and the Principle 9—Individual Access support rights to consent (Section  of Schedule 1), access, correction, and deletion “depending upon the nature of the information challenged” (Section 4.9 of Schedule 1). The Principle 2—Identifying Purposes and the Principle 8—Openness support an individual’s right to transparency regarding the collection purposes and the organizational privacy practices. And the Principle 10—Challenging Compliance gives individuals the right to file complaints and challenge an organization’s practices.

As discussed previously, personal information is arguably “used” when generating and evaluating synthetic data. However, the legal basis of an organization for such use, whether it requires consent or is permitted without consent, is not clear under PIPEDA. This is true with respect to both the organization that has custody of the real data, as well as any third party to which the organization may transfer the personal information to conduct these activities on its behalf. If consent is not required for these activities, the individual cannot have a right to withdraw their consent for these purposes. However, it is important to note that other FIPs, including the individual privacy rights as outlined above, would continue to apply when personal information is used for SDG.

When it comes to the synthetic data itself, the applicability of individual rights depends entirely on whether this data qualifies as personal information under PIPEDA. If synthetic data is considered as non-personal information, then individuals have no rights to access, correct or delete synthetic data because these rights are limited to personal information (i.e., information about an identifiable individual). However, if the residual risks are too high, then it would still fall under the scope of PIPEDA. Enforcing such rights directly on synthetic data presents serious challenges and an organization would need to reverse its SDG process to give effect to these rights. This, apart from being technically challenging, is, in and of itself, a privacy invasive activity that impacts the policy objectives of privacy laws. Thus, in such cases, the proper application of these rights must occur at the stage where the original personal information is used to generate synthetic data, rather than with respect to the synthetic data itself.

## Discussion

### Summary

This study aimed at identifying privacy gaps for SDG, its use and disclosure under the current Canadian federal privacy regulation PIPEDA. As SDG or synthetic data is not an explicit part of current legislation, the questions were addressed considering SDG as a process of reducing the identifiability of data. This is in line with global regulatory authorities who have drafted guidelines on SDG as another type of PET [33–35].

Our interpretation of SDG as a “use” of personal information is confirmed by accumulating legal opinions on SDG under other jurisdictions. For such “use” (or “processing”), requirements set out in the respective privacy statutes apply that typically involve a lawful basis for the use of personal information. Consent may not necessarily be required when making the case of legitimate interest under, for example, the GDPR. Under PIPEDA, the question regarding consent is more challenging given the limited set of permitted purposes for using personal information without consent. While it is reasonable from a purpose-based perspective that SDG and related technologies like de-identification are treated as permitted uses under PIPEDA, uncertainty remains whether or not consent is necessary for SDG.

The resulting synthetic data can be outside the scope of PIPEDA if it qualifies as non-personal information. While under more recent regulatory frameworks such as the ones in Europe or Quebec, identifiability encompasses a simultaneously broader and more precise concept with three criteria, identifiability under PIPEDA has historically mainly centered around identity disclosure only. Identity disclosure should be protected by SDG by design (no one-to-one mapping), which is why synthetic data may have a greater potential to qualify as non-personal than more traditional data perturbation methods under PIPEDA. While inferences as a privacy violation are increasingly discussed and demanded in other regulatory contexts, it remains unclear if identifiability under PIPEDA can be interpreted to encompass the criterion of inferences as well. As a practical matter thus far, identifiability may therefore reasonably be interpreted narrowly under PIPEDA, focusing primarily on identity disclosure.

### Recommendations for a future framework

Our analysis shows that the degree to which the questions are addressed under the current federal statute in Canada varies. In the following, we discuss potential risks that arise from the identified gaps and give concrete recommendations on how to mitigate them in future regulatory frameworks. A general principle is that privacy regulation should incentivize the adoption of PETs so that organizations are encouraged to apply PETs to achieve data minimization and, ultimately, protect an individual’s privacy. Privacy statutes can be improved not only to support organizations in meeting this goal more effectively, but also to increase regulatory certainty and reduce the risks privacy professionals are taking when relying on their own interpretations to navigate regulatory ambiguities.

Whether the use of the original (real) dataset to generate and/or evaluate a synthetic dataset is regulated and, in particular, whether consent is required for such processing of personal information remains ambiguous under PIPEDA. The statute does not define the term “use” and unlike, for example, Ontario’s PHIPA [64], it does not explicitly recognize reducing the identifiability of data as a permissible process where personal information can be used without knowledge and consent. Organizations may consequently be reluctant to invest in SDG as a PET and may instead continue to process personal information directly. To address this gap, future frameworks should clarify that an organization may use personal information without the individual’s knowledge and consent for the purpose of reducing the identifiability of the information, and to evaluate the effectiveness of this process. This would provide more legal certainty while being consistent with the technology-agnostic approach of the statute.

While PIPEDA includes transparency obligations through various FIPs, there is limited guidance on how to apply them in the context of SDG. Individuals may therefore not understand or be unaware of how their personal information is used to create synthetic data. In practice, organizations should document the processes they have undertaken to reduce the identifiability of the data and to evaluate the effectiveness of this process. They should be required to be transparent that they use personal information to generate synthetic data, how this process works in general terms, how the resulting synthetic dataset is used and/or disclosed and what rights apply (or no longer apply) to the resulting synthetic dataset. In this context, Privacy Impact Assessments (PIAs) can also serve as an important instrument not only for identifying and mitigating privacy risks but also for promoting transparency through documentation. While PIAs are not mandated under PIPEDA, they have been recommended as a requirement for organizations, for example, in the OPC 2020 recommendation on the regulation of AI [66].

Similarly, there is limited guidance on the rights and responsibilities of both, organizations that engage with third-party service providers for SDG and the third-party service providers themselves. In practice, most organizations do not have the in-house capabilities necessary to create and evaluate synthetic data and will therefore need to engage the services of an expert third party to conduct these activities on its behalf. With the residual uncertainty in current legislation, one risk is that a third party would not be involved. This could result in improper SDG if organizations do not have the in-house capabilities, or in the avoidance of SDG altogether. Even where organizations do engage a service provider, there is a risk that, without clear statutory direction, contracts will fail to provide meaningful protection or accountability. To address this, regulation should clearly define the term “service provider” as “an organization, including a parent corporation, subsidiary, contractor or subcontractor, that provides services for or on behalf of another organization for which the service provider requires access to the personal information maintained by the organization and subject to the purposes for which the organization may collect, use and/or disclose under this Act”. It should confirm that an organization is accountable for personal information under its control and the meaning thereof, that the obligation of the organization is to ensure by contractual or other means that the service provider provides substantially the same protection of the information as does the organization, that the service provider is limited to using and disclosing the personal data as set out in the contract for the purposes of the organization and not for its own purposes, that knowledge and consent is not required for the process of transferring personal information to a service provider and that the service provider has the obligations to comply with the provisions related to security safeguards and breach notification.

Whether PIPEDA regulates the resulting use and disclosure of the synthetic dataset is unclear as the statute does not define identifiability nor provides requirements for rendering data non-personal. In the absence of legal criteria, organizations may interpret non-personal information as data with zero disclosure risk. This can result in situations where legal exposure and complexities are avoided and promising technologies such as SDG fail to be broadly adopted [97–99], or, at the other extreme, situations where identifiability is not measured at all, or non-identifiability is incorrectly claimed which jeopardizes not only individuals’ privacy but also trust in PETs. As a general principle, organizations should be encouraged to adopt the highest standard possible. In the current regulatory regime, it is mainly through a reactive lens (e.g., privacy complaint or an access to information request) that an organization will find out whether its actions to create non-personal information met the standard according to a privacy regulator. On the other hand Canadian regulators have significant concern that de-identification may not always be applied properly [65]. Therefore, key stakeholders are uncertain about whether actual practices to reduce identifiability are sufficient.

To address these gaps, identifiability should be defined in line with the “reasonably likely” standard, and it should be recognized that non-personal information may still involve a very small residual risk. Criteria for identifiability should be established, encompassing not only traditional identity disclosure risk but also risks from inferences. However, a legal definition, even if more precise, does typically not have the necessary details (e.g. best practices, thresholds, metrics) to provide clear guidance for proper privacy-protective SDG. Such guidance can be provided by referring to good practices, guidelines or Codes of Practice. Such an approach would allow for flexibility in legislation so that it remains applicable as technology and societal contexts evolve, while providing a more enforceable set of guidelines [65]. A Code of Practice anchored in the applicable privacy legislation could give necessary assurance to regulators but also more certainty to organizations who want to implement PETs such as SDG. It also allows for a comprehensive and flexible way to account for the changing landscape of an evolving technology, and to ensure consistency across organizations. A certification system as proposed in [44] could then be a transparent process to give official recognition that an approach to reduce identifiability follows best practices thereby providing certainty for organizations and regulators. A Code of Practice can be based on standards such as the International Standard on Information security, cybersecurity and privacy protection—Privacy enhancing data de-identification framework (ISO/IEC 27559) [86] or locally implemented guidelines such as the De-Identification Guidelines for Structured Data by the Information and Privacy Commissioner of Ontario [85]. There are, however, only few regulators who have published guidance on SDG so far [33–35] and no international standard is available. While general concepts of risk-based de-identification also apply to SDG, major privacy risks center around inferences and not identity disclosure and are consequently different from those in data-modifying de-identification. It can be expected, however, that with accumulating evidence and experience more comprehensive guidance documents will follow, so that these could be referenced in future regulations.

### Limitations

This study gives detailed insights into the Canadian federal private sector privacy regulation as it relates to SDG, synthetic data use and disclosure. The following limitations must be indicated:

1. This study refrained from analyzing Bill C-27 as it is still in the legislative process and is very likely to be subject to further adjustments.
2. Our work focuses on Canadian federal private sector privacy regulation. Other jurisdictions come with different gaps, but generic themes were identified and described in recommendations that are applicable across jurisdictions.
3. We focused on selected questions around SDG, synthetic data use and disclosure. These questions have been relevant in evaluations under other legal frameworks as well [19, 38–41] and include practical considerations that commonly arise in real-world SDG implementations. There may be further questions of interest that warrant in-depth exploration.
4. This study did not delve into privacy metrics and practices for synthetic data. While privacy metrics for synthetic data have been proposed [28], it is important to highlight that performance can substantially vary across and also within model classes depending on the dataset and use case [100, 101]. This suggests that there is no SDG model-specific superiority that would support a meaningful identification of particularly useful models. As a result, evaluations should be based on the very synthetic dataset that is disclosed. Such an approach is consistent with PIPEDA, which regulates the handling of personal information rather than the PETs themselves.

Ultimately, this paper focused on privacy and regulatory oversight which represent critical dimensions to ensure fair and responsible data use and disclosure. However, concerns that are orthogonal to data identifiability such as bias must also be addressed. It is also important to acknowledge that where regulatory uncertainty persists, broader normative frameworks may step in to guide decision-making. Such broader frameworks are reflected, for example, in the UK Information Commissioner’s Office anonymization guidance [102] which emphasizes that anonymous information should be used in ways that people would reasonably expect and to avoid or justify any adverse impacts. Similarly, South Korea’s guidelines for SDG [103] includes an oversight board that applies expert judgment on the safety of synthetic data and in multiple countries, research ethics boards are evaluating and approving the use of data in research. Moreover, PIAs, while primarily regulatory instruments, can also support decision-making where regulatory requirements are ambiguous. These examples show that broader ethical frameworks are embedded in institutional processes and can help fill regulatory gaps.

## Data Availability

The Canadian federal privacy law analyzed in this paper is available to the public under: https://laws-lois.justice.gc.ca/eng/acts/P-8.6/index.html.
